# Bio-inspired solutions to the challenges of chemical sensing

**DOI:** 10.3389/fneng.2012.00024

**Published:** 2012-10-29

**Authors:** Ramon Huerta, Thomas Nowotny

**Affiliations:** ^1^BioCircuits Institute, University of CaliforniaSan Diego, CA, USA; ^2^CCNR, Informatics, University of SussexBrighton, UK

It is widely recognized that further breakthroughs in science and technology may rely on multidisciplinary research efforts. Breaking the boundaries of well-established research fields and combining methodologies from disparate areas can foster innovative and translational research. Chemical sensing is no exception. Perhaps more than any other sensory modality, chemical sensing is plagued with major technical and conceptual challenges: the turbulent nature of the signal carrier, the long term instability, lack of sensitivity, and slow response times of sensors and the lack of a reliable odor map to characterize mammalian perception. When facing these hurdles, the designers of artificial devices for gas recognition look at the olfactory system of animals for inspiration because animals seemingly effortlessly accomplish some of the unsolved challenging problems in machine olfaction: recognizing odors and odor mixtures from a chemical background, segmenting mixtures of odors into components, being sensitive and robust and extracting the same odor percept over a wide range of concentrations.

Our challenge in bio-mimetic chemical sensing is to identify at all levels from the olfactory receptors to the central nervous system, what are the key ingredients to these impressive abilities. The goal of this research topic is to document highlights from this ongoing effort and to compile an up-to-date overview not only from the academic point of view but also with respect to industrial applications.

This research topic emanates from our own effort to bridge the anatomical and physiological data of the olfactory system, in particular in insects, to explain pattern recognition (Huerta et al., 2004; Nowotny et al., 2005; Huerta and Nowotny, 2009) and apply them to real problems with artificial sensor arrays and other applications (Muezzinoglu et al., 2008, 2009; Huerta et al., 2012). This research topic therefore brings together researchers from chemistry, neuroscience, physics, biology, and computer science, and the described work extends from fundamental scientific questions to technological applications.

On the scientific side the contributions tackle three core issues: concentration-invariant representations of odors, properties, and the potential role of oscillations in the olfactory system and the nature of odor interactions in mixtures.

Cleland et al. (2012) address concentration-invariant odor perception in rats and find that there are six known mechanisms that combine to achieve odor representations that do only minimally depend on concentration. Yamani et al. (2012) take a different view on concentration-invariant odor perception. Taking inspiration from the convergence of olfactory receptor neurons onto glomeruli and the use of latency as the coding signal they design a bio-mimetic information processing method for a metal oxide gas sensor array. Martinelli et al. (2011) on the other hand have identified another advantage of latency coding. They propose a bio-inspired solution to accelerate the response to odors using a network that uses the spike latency to discriminate volatile gases.

The presence of clear but transient oscillations in many olfactory systems has baffled scientists for a long time. The review of Assisi and Bazhenov (2012) summarizes their extensive work on the origin of these oscillations. However, Daly et al. (2011) report that they discovered that in the hawk moth *Manduca Sexta* oscillatory patterns have quite different characteristics than previously reported: oscillations are frequency modulated by odor input rather than amplitude-modulated, they are local rather than global and spikes lock to the oscillations during baseline activity more than during odor stimuli. Schmuker et al. (2011) address the problem of concurrent odor recognition and odor concentration estimation using models of the bee olfactory system and demonstrate that either goal can be achieved by the same antennal lobe network depending on the strength of lateral inhibition. In a similar direction, Proske et al. (2012) investigate different network topologies in an antennal lobe model of the fruit fly *Drosophila* and find that networks with a heterogeneous, correlation based inhibitory network lead to the best discrimination performance. Capurro et al. (2012) address the problem of odor interactions in odorant blends. Comparing experimental data from local and projections neurons with a reduced model they conclude that the experimentally observed odor interactions can be explained solely through local neuron and projection neuron response profiles and a simple mechanism of lateral inhibition.

On the technological side we are pleased to have received a variety of articles describing applications of chemical sensors and electronic noses, robotics, and a clinical application for hyposmia/anosmia characterization (Yáñez et al., 2012).

Vergara and Llobet (2012) address the problem of better selectivity on sensor technology using signal modulation which is directly related to the oscillatory behavior investigated in the biological systems above. In robotic implementations of gas sensing, Hernandez Bennetts et al. (2012) compare biologically plausible models and statistical strategies for odor localization. Their results are not too optimistic because, as they argue, current sensor technology still differ fundamentally in their sensing and actuation capabilities from highly sensitive and fast biological chemical receptors. It is clear that we still need to develop more sensitive, faster, and more stable sensors to be able to take full advantage of bio-inspired technology here. Nevertheless, in the meantime, until this technology is fully developed there are alternative hybrid approaches that allow testing strategies with simulated gas sensor responses and real robots as described in this research topic by Rhodes and Anderson (2012). This idea may point out a direction in which multidisciplinary approaches can advance in seeking the prospects of the neuro-inspired algorithms for information processing.

In summary this special issue is providing an overview of the frontiers in olfactory processing in the brain and the current challenges in applying these neuro-inspired principles in artificial olfaction. Much progress has been made, and it is becoming clear that the path to future breakthroughs does not emerge from isolated fields, but from multidisciplinary efforts like the ones presented here.
